# Supplementary material for: Optimal methods for analyzing targeted pairwise knockout screens
Source: bioRxiv. 2024 Aug 20:2024.08.19.608665. Preprint. [Version 1] doi: 10.1101/2024.08.19.608665 (PMC11370406; doi:10.1101/2024.08.19.608665)
Supplement: Supplement 2 [file NIHPP2024.08.19.608665v1-supplement-2.pdf]

bioRxiv preprint doi: https://doi.org/10.1101/2024.08.19.608665; this version posted August 20, 2024. The copyright holder for this preprint (which was not certified by peer review) is the author/funder, who has granted bioRxiv a license to display the preprint in perpetuity. It is made available under aCC-BY 4.0 International license.

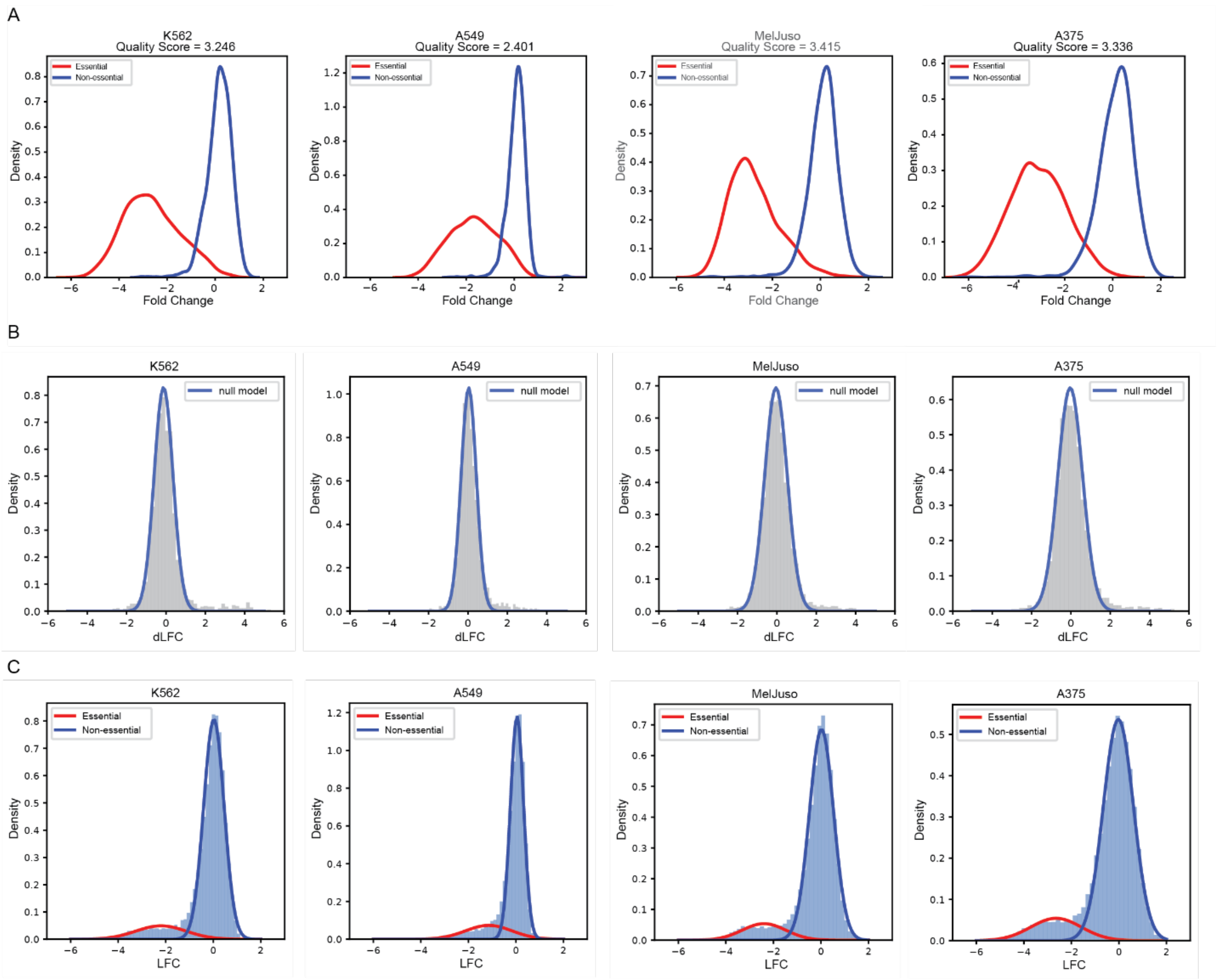


**Supplementary Figure 1.** (A) Fold change distributions of arrays targeting reference essential (red) and non-essential (blue) genes, along with Cohen's D quality score in four cell lines. This includes the prototype library in K562 and A549 cell lines, and the Inzolia library in MelJuso and A375 cell lines. (B) The dLFC histograms of the four cell lines with normal distribution fits after removing outliers (Methods). The blue curves represent the fit of the null model. (C) LFC histograms of the four cell lines, with a two-component Gaussian Mixture model representing the distribution. The red component models the essential genes, while the blue component represents the majority of genes that do not show severe fitness defects.

bioRxiv preprint doi: https://doi.org/10.1101/2024.08.19.608665; this version posted August 20, 2024. The copyright holder for this preprint (which was not certified by peer review) is the author/funder, who has granted bioRxiv a license to display the preprint in perpetuity. It is made available under aCC-BY 4.0 International license.

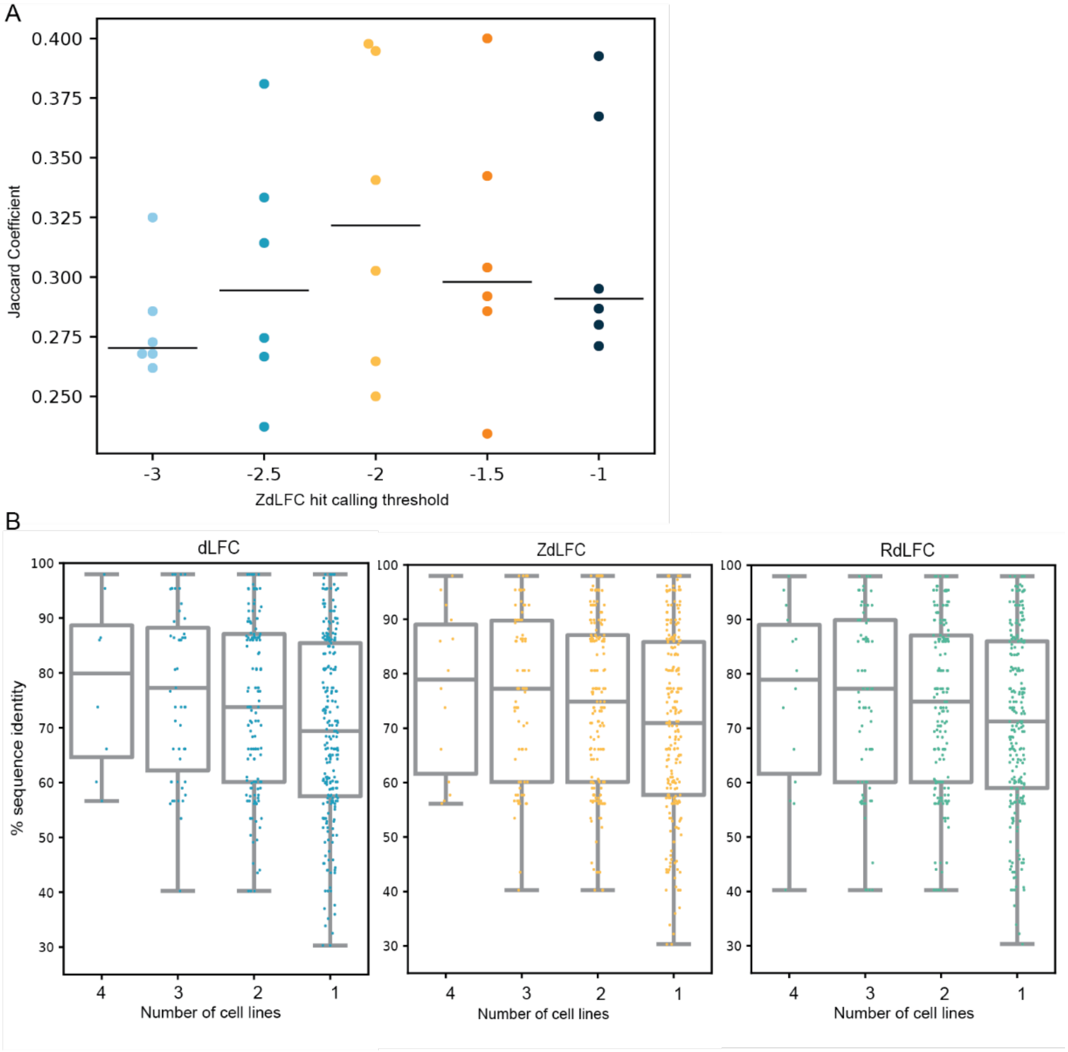


**Supplementary Figure 2.** (A) Jaccard coefficients showing the hits across all pairs of cell lines with different thresholds using the ZdLFC method. Black line indicates the median Jaccard coefficient for each threshold. (B) Swarm plots combined with box plots showing the percent sequence identity of all synthetic lethal pairs identified in all four, three, two, and one screens using the three methods.
